# Pediatric ovarian sliding inguinal hernia, is surgical repair urgent?

**DOI:** 10.15537/smj.2022.43.9.20220492

**Published:** 2022-09

**Authors:** Saud A. Al Jadaan, Mutaz M. Gieballa, Suliaman M. Alaqeel, Mohammad A. Aldaffaa

**Affiliations:** *From the Department of Pediatric Surgery (Al Jadaan, Gieballa, Alaqeel, Aldaffaa), King Abdullah Specialized Children’s Hospital, King Abdulaziz Medical City; from the College of Medicine (Al Jadaan, Gieballa), King Saud bin Abdulaziz University for Health Sciences; and from King Abdullah International Medical Research Center (Al Jadaan, Gieballa, Alaqeel), Riyadh, Kingdom of Saudi Arabia.*

**Keywords:** indirect inguinal hernia, sliding hernia, ovary, incarceration

## Abstract

**Objectives:**

To find if repairing sliding inguinal hernias containing the ovary should be carried out urgently or not.

**Methods:**

A retrospective chart review carried out at King Abdulaziz Medical City, Riyadh, Saudi Arabia from 2015-2022. All cases with an ovarian inguinal hernia in females aged 0-14 years were reviewed after obtaining approval from the Institutional Review Board.

**Results:**

Female children with inguinal hernia patients were 191. A total of 28 cases were sliding inguinal hernias involving the ovary. All were repaired electively except for 4 cases that had an initial presentation of an incarcerated ovary at age less than 90 days which required urgent reduction and repair.

**Conclusion:**

Based on our review, ovarian sliding hernias in female patients can be repaired electively, although newborns are at a higher risk of complications from sliding inguinal hernias.

Inguinal hernias are a commonly encountered entity by pediatric surgeons. The overall estimated incidence is approximately 1-5%.^1^ Female inguinal hernias represent 20-23% of all hernia cases.^2,3^ As per Bendavid’s^4^ definition, a sliding hernia is formed when a retroperitoneal organ protrudes outside the abdominal cavity in such a manner that the organ itself and the overlying peritoneal surface constitute a side of the hernia sac. Sliding inguinal hernias in females are more common than in males. Sliding inguinal hernias have been observed in 15-45% of inguinal hernia in females; ovary being the most common organ.^3^ The timing and urgency of ovarian sliding hernia repair in females have not been standardized and remain debatable. We carried out a retrospective review in an attempt to address whether the timing of repair is urgent or not.

## Methods

A retrospective chart review analysis of female inguinal hernia with a sliding component cases in King Abdulaziz Medical City, Riyadh, Saudi Arabia, from 2015-2022 was carried out. This study was initiated after obtaining ethical approval from King Abdullah International Medical Research Center (KAIMRC), Riyadh, Saudi Arabia (reference No.: RC19/303/R). Also, this study was carried out with the ethical standards of the National Guard Health Affairs and KAIMRC. All the data collection forms were kept under strict confidentiality, accessible only to the data collectors.

After obtaining the medical record numbers, charts were reviewed from electronic records and data were entered in an electronic data collection sheet. Any patient with urogenital disorders or ambiguous genitalia were excluded.

The data collection sheet included the age of diagnosis, the site of hernia (right, left, or bilateral), clinical presentation, timing of surgery, ultrasound findings, intra-operative finding, and number of post-operation (op) visits.

### Statistical analysis

The Statistical Package for Social Science, version 22.0 (IBM Corp., Armonk, NY, USA) was used for data analysis. Mean and standard deviation (SD) were used for numerical data and frequencies used for categorical data. Chi-square test was used to compare between data categories. A *p*-value of <0.5 was considered significant.

## Results

A total of 191 females with inguinal hernias that were diagnosed and repaired between 2015-2022. Ovarian sliding inguinal hernia was observed in 28 (14.6%) cases (Table 1). All cases were females without urogenital anomalies or ambiguous genitalia. The mean age of diagnosis of ovarian sliding hernia was 6.6±9 months (range: 25 days - 3 years). A total of 24 (85.7%) of 28 were repaired on an elective basis. Elective repair was carried out 0-448 days after diagnosis with a mean of 44.25 days. Four (14.3%) of 28 cases initially presented with an incarcerated hernia that were diagnosed by clinical and radiological findings, and all were aged less than 90 days with a mean age of 49±16 days (range: 28-84 days). All patients were treated by pediatric surgeons. In all cases, the type of repair was the standard inguinal open approach with closure of the sac using an absorbable purse-string suture.

**Table 1:** - Sliding inguinal hernia characteristics.

Variables	n (%)
***Site***	
Right	18 (64.0)
Left	7 (25.0)
Bilateral	3 (11.0)^†^
Organ involved*	
Fallopian tube	9 (31.0)
Uterus	1 (4.0)
Appendix	1 (4.0)
Omentum	1 (4.0)
***Age at repair***	
<90 days	16 (56)
>90 days	12 (43)

Values are presented as a number and percentage (%). *Along with ovary, ^†^sliding component is only one side

Four underwent immediate repair after being diagnosed clinically and radiologically as incarcerated hernia containing ovary with 4 being non-reducible. No statistical significance was found between ovary presence in the hernia vs urgency of repair (*p*=0.0978). For the site of hernia, 18 patients were right sided, 7 left sided, and 3 were bilateral. For the bilateral cases, the inguinal hernia with a sliding component was only unilateral. Three of the right sided were carried out urgently, none of the left sided were urgent. One of the bilateral underwent immediate repair for right side hernia containing ovary with the left side repaired in the same setting. There was no statistical significance between site of hernia and urgency of repair (*p*=0.258).

All our patients had an ovary with or without fallopian tube/uterus as part of the inguinal hernia sac. All were examined intra-operitevly for necrosis, ischemia, or torsion. All patients had a normal ovary at the time of repair. Four of those were operated urgently because they were irreducable. The viability of the ovaries was analysed in this study. The intraoperative finding and the ultrasound at the time of diagnoses were normal in 28 of these patients. Also no emergency room visits afterwards and all had normal findings on post-op outpatient department visits (one visit). The relationship between the viablity of the ovary and the urgency of repair was not statistically significant (*p*=0.1429).

## Discussion

Sliding inguinal hernia is more commonly seen in females than males.^2,3^ The most common organ in sliding inguinal hernia is the ovary, although the fallopian tube or the uterus may also occur.^3,5^ The timing of sliding inguinal hernia repair is still controversial among pediatric surgeons, and strong evidence is still lacking. In our review, the incidence of sliding inguinal hernia among females was 28 (14.6%), approximately 85.7% were repaired electively, and approximately 14.3% of the cases presented with incarceration/strangulation, and it was their initial presentation of inguinal hernia. Incarceration and strangulation were unavoidable in these 4 cases as there have been no prior diagnosis of hernia in these patients. All patients who presented with incarceration/strangulation were aged less than 90 days. The diagnosis of incarceration/strangulation was based on clinical and ultrasound with doppler flow study findings.

Several studies have reviewed sliding inguinal hernia in females. Boley et al^2^ reviewed 68 cases of inguinal hernias in females, including 19 sliding inguinal hernias. Although the authors recommended prompt repair, they did not report any complications prior to repair. Also, another study carried out by Huang et al^6^ included 26 sliding inguinal hernia cases that presented with an asymptomatic palpable inguinal mass, and there was no report of complications before repair in their review. Furthermore, a prospective multicenter study in Nigeria carried out by Osifo et al^7^ that included 138 female inguinal hernia cases with 8.4% that were irreducible, in their study, all repair was carried out on an elective basis and there was no report of strangulation or torsion. On the other hand, Takehara et al^8^ reviewed the role of laparoscopy in females with irreducible inguinal hernia, which included 46 cases of sliding inguinal hernias. Four out of 46 presented with incarcerated hernia while waiting for elective repair. All were carried out using the open technique, 3 cases being under a year old.

Sliding inguinal hernia is more common in females aged less than one year compared to those older than one year.^5^ Apart from age, Chen et al^9^ attempted to review risk factors associated with developing strangulated inguinal hernias in females and found that ovarian volume over 5 cm was associated with more risk of strangulated ovarian hernia. Therefore, torsion may be an important factor in the mechanism of ovarian necrosis in sliding ovarian hernia.

### Study limitations

A small sample size, retrospective nature of the study, and a single institution study. Also the post-op visits was limited to one visit.

In conclusion, this study shows that sliding inguinal hernia containing an ovary can be safely repaired electively. However, if a patient is diagnosed at an age younger than 90 days of age, repair should be carried out urgently due to the risk of incarceration, strangulation, or torsion. Prospective studies with a larger sample size are needed to confirm our conclusion.
